# A 4-Site Public Deliberation Project on the Acceptability of Youth Self-Consent in Biomedical HIV Prevention Trials: Assessment of Facilitator Fidelity to Key Principles

**DOI:** 10.2196/58451

**Published:** 2025-02-13

**Authors:** Claire Burke Draucker, Andrés Carrión, Mary A Ott, Ariel I Hicks, Amelia Knopf

**Affiliations:** 1 Indiana University Indianapolis, IN United States; 2 Icahn School of Medicine at Mount Sinai New York, NY United States

**Keywords:** public deliberation, deliberative democracy, bioethics, ethical conflict, biomedical, HIV prevention, HIV research, group facilitation, fidelity assessment, content analysis

## Abstract

**Background:**

Public deliberation is an approach used to engage persons with diverse perspectives in discussions and decision-making about issues affecting the public that are controversial or value laden. Because experts have identified the need to evaluate facilitator performance, our research team developed a framework to assess the fidelity of facilitator remarks to key principles of public deliberation.

**Objective:**

This report describes how the framework was used to assess facilitator fidelity in a 4-site public deliberation project on the acceptability of minor self-consent in biomedical HIV prevention research.

**Methods:**

A total of 88 individuals participated in 4 deliberation sessions held in 4 cities throughout the United States. The sessions, facilitated by 18 team members, were recorded and transcribed verbatim. Facilitator remarks were highlighted, and predetermined coding rules were used to code the remarks to 1 of 6 principles of quality deliberations. A variety of display tables were used to organize the codes and calculate the number of facilitator remarks that were consistent or inconsistent with each principle during each session across all sites. A content analysis was conducted on the remarks to describe how facilitator remarks aligned or failed to align with each principle.

**Results:**

In total, 735 remarks were coded to one of the principles; 516 (70.2%) were coded as consistent with a principle, and 219 (29.8%) were coded as inconsistent. A total of 185 remarks were coded to the principle of equal participation (n=138, 74.6% as consistent; n=185, 25.4% as inconsistent), 158 were coded to expression of diverse opinions (n=110, 69.6% as consistent; n=48, 30.4% as inconsistent), 27 were coded to respect for others (n=27, 100% as consistent), 24 were coded to adoption of a societal perspective (n=11, 46% as consistent; n=13, 54% as inconsistent), 99 were coded to reasoned justification of ideas (n=81, 82% as consistent; n=18, 18% as inconsistent), and 242 were coded to compromise or movement toward consensus (n=149, 61.6% as consistent; n=93, 38.4% as inconsistent). Therefore, the counts provided affirmation that most of the facilitator remarks were aligned with the principles of deliberation, suggesting good facilitator fidelity. By considering how the remarks aligned or failed to align with the principles, areas where facilitator fidelity can be strengthened were identified. The results indicated that facilitators should focus more on encouraging quieter members to participate, refraining from expressing personal opinions, promoting the adoption of a societal perspective and reasoned justification of opinions, and inviting deliberants to articulate their areas of common ground.

**Conclusions:**

The results provide an example of how a framework for assessing facilitator fidelity was used in a 4-site deliberation project. The framework will be refined to better address issues related to balancing personal and public perspectives, managing plurality, and mitigating social inequalities.

## Introduction

### Background

Public deliberations are community-focused activities in which persons with diverse perspectives are engaged in discussion and decision-making about a topic that is controversial or value laden or involves ethical uncertainty. Public deliberations typically include provision of factual and balanced information about the deliberation topic, facilitated small- and large-group discussions, and activities that yield collective solutions to policy questions [1-3].

Public deliberation has been used to explore a wide variety of issues that affect public health. The method has been used to elicit views on genetic research and testing in Alaska Native and American Indian communities [4], attitudes and recommendations to address antibiotic overuse [5], opinions on acceptable uses of deidentified health information in oncology settings [6], views on the allocation of limited health care resources [7], opinions on guidelines for the design and evaluation of decision aids [8], recommendations for opioid screening in the context of HIV care [2], preferences regarding health data sharing for patients with cancer [9], and views on precision medicine research in American Indian and Alaska Native communities [10].

With the increasing use of public deliberations to inform public health policy decisions, experts have called for increased attention to assessing and reporting the quality of deliberations [11,12]. To aid in this task, Scott et al [11] developed a deductive coding framework based on the operationalization of common deliberation goals and a synthesis of published frameworks designed to assess deliberation quality. Their framework includes 4 deliberation elements (ie, preferences and values are referenced, deliberants engage with each other, expert information is referenced, and information enriches deliberation) and 4 recommendation elements (ie, recommendations are clear and identifiable, recommendations are relevant, recommendations are justified, and recommendations reflect a societal perspective). Goold et al [12] also developed an analytic framework for the assessment of deliberation quality. This framework calls for an evaluation of structure (eg, demographic representativeness of the deliberants), process (eg, equality of participation), and outcomes (eg, deliberant knowledge of deliberation topics) using a variety of data sources and analytic approaches (eg, surveys, follow-up interviews, qualitative analysis of transcripts, word counts, and participation metrics).

Most evaluation frameworks do not provide for an assessment of the performance of facilitators. Deliberations typically rely on skilled facilitators who are responsible for promoting deliberative norms, fostering high-quality discussion, and promoting an outcome of shared solutions. Rubinelli and von Groote [13] outline common facilitator tasks, including conducting the dialogue, soliciting different opinions, ensuring that reasons are provided for points of view, and ensuring that reasons are grounded in evidence. The authors also discuss facilitator challenges such as identifying hidden goals of deliberants and managing sharp differences of opinion. Dillard [14] argues that facilitators are “the first and primary form of intervention” and provides an in-depth discussion of facilitator responsibilities. These responsibilities can include setting the discussion tone, establishing deliberative rules and norms, mitigating adversarial and polarizing dynamics, and ensuring that minority opinions are expressed. Moreover, the author identifies 3 facilitator types based on variations in their actions: passive facilitators (ie, they regulate deliberant speech time and direct participant turn taking), involved facilitators (ie, they ask elicitation questions and implement discursive strategies), and moderate facilitators (ie, they use elicitation questions and discursive strategies but less often than involved facilitators). Discursive strategies are facilitation techniques used to manage tensions and encourage fruitful deliberative discourse. These strategies include storytelling (ie, sharing personal experiences), playing devil’s advocate, framing (ie, highlighting interactions to shape discussion), focusing on local issues to consider specific implications, and eliciting responses.

### Objectives

Despite the assumed importance of facilitators in ensuring effective deliberative processes, published reports of deliberation outcomes rarely include information regarding facilitator credentials, selection, training, and supervision and, with few exceptions (see the study by De Vries et al [15]), do not address the quality of facilitator involvement. Therefore, a framework is needed to guide researchers in systematically assessing public deliberation facilitator remarks. To address this gap, our research team developed such a framework. This framework is based on the assumption that the performance of facilitators can be assessed by determining the fidelity of their remarks to well-established principles of democratic deliberation. A blueprint to guide researchers in implementing the framework has been published in *JMIR Formative Research* [16] and includes several coding templates and a step-by-step analytic plan. The purpose of this report is to describe how the framework (hereafter referred to as the blueprint report) was used to assess facilitator fidelity in our 4-site public deliberation on the acceptability of youth self-consent in biomedical HIV prevention research. In this report, we (1) briefly describe the deliberation, (2) summarize the blueprint report, and (3) present our assessment of facilitator fidelity across the 4 sites.

## Methods

### Public Deliberation

The deliberation research project from which this work stemmed is described in depth elsewhere [17,18] and summarized in the blueprint report [16]. Therefore, we include only a brief recap of the main aspects of the deliberation project in this section.

The public health problem that was the focus of the deliberation was the underrepresentation of minors in biomedical HIV prevention trials due in part to barriers caused by standard regulatory requirements for parental consent and youth assent [19,20]. This consent model can result in parents becoming aware of some stigmatized behaviors or identities that their children may not wish to disclose and, thus, can lead to rejection or punishment of the youth or their exclusion from research [21]. Because youth from underrepresented populations assume a disproportionate burden of HIV disease and are the most likely to be harmed by existing consent processes, calls have been made for consideration of new consent procedures, including minor self-consent [22]. However, because parental consent has long been considered foundational to protecting youth from research-related harms, developing new consent models constitutes a public health issue that is both controversial and value laden. The purpose of our deliberation was to engage diverse stakeholders, including minor adolescents, parents, caregivers, and community members, in deliberations about the use of minor self-consent in biomedical HIV prevention research.

### Ethical Considerations

This study was reviewed and approved by the Indiana University Institutional Review Board (IRB; protocol 2001909245), which served as the single IRB in accordance with reliance agreements from participating institutions, including the University of South Florida, Johns Hopkins University, the University of Colorado Denver, and the University of Chicago. An electronic copy of the IRB-approved study information sheet was provided to deliberants by study staff or site investigators. In web-based or face-to-face meetings, staff reviewed study information with interested deliberants and obtained verbal consent from those who agreed to participate. Although a waiver of parental permission was granted for this study, minors were asked whether they felt comfortable seeking parental permission to enroll in the study. Those who wished to engage a parent helped staff schedule a time to meet with both the minor and their parent or guardian to review the study information sheet and obtain parental permission. If a minor did not wish to engage a parent, the study staff contacted a site principal investigator, who determined whether the waiver of parental permission could be applied.

All participants were assigned a study ID number, and their files were labeled with that ID number. All data were stored on a dually authenticated, password-protected, cloud-based storage system. The servers are tightly controlled and inaccessible to anyone without appropriate credentials. Once all analyses are complete, audio and video files will be permanently deleted, and only deidentified data will be stored.

Participants received compensation for this study, which varied by activity and retention in the study. The maximum compensation for participants who completed all surveys and homework and attended all deliberative sessions was US $300 in the form of gift cards from a variety of web-based and physical stores; participants selected the retailer that worked best for their needs.

### Deliberation Sessions

#### Overview

The deliberations were held via a web-based video platform in 4 cities: Tampa, Florida; Baltimore, Maryland; Denver, Colorado; and Chicago, Illinois. Educational materials related to the deliberation topic were developed in consultation with a youth advisory board and external advisers and displayed on a deliberant website. At each site, 4 deliberation sessions, each lasting 2 hours, were held. Each session included large-group meetings (plenary sessions) and small breakout group sessions. During the plenary sessions, research team members and expert stakeholders (eg, physicians and IRB administrators) presented information on topics pertinent to the deliberation (eg, research with adolescents, regulatory guidelines for biomedical research, and clinical trial principles). All deliberation sessions were video recorded and transcribed. Due to recording malfunctions in Baltimore, transcripts were not available for some of these sessions.

#### Deliberants

There were 88 deliberants in the deliberations. To provide context for our discussion on facilitator fidelity, a summary of the deliberants’ demographic characteristics is presented in Table 1.

**Table 1:** Summary of deliberants’ demographic characteristics (N=88).

Characteristics		Deliberants, n (%)
**Age**		
	Minor	24 (27)
	Young adult	20 (23)
	Adult	44 (50)
**Gender**		
	Women, including transgender women and transfeminine	62 (70)
	Genderqueer or gender nonconforming	6 (7)
	Men, including transgender men and transmasculine	17 (19)
	Other	3 (3)
**Sexual orientation**		
	Lesbian, bisexual, gay, or queer	26 (30)
	Heterosexual or straight	53 (60)
	Questioning	5 (6)
	Other^a^	4 (5)
**Educational level (n=60)^b^**		
	High school diploma or GED^c^	8 (13)
	Some college or technical program	12 (20)
	Finished vocational or technical program or 2-year degree	5 (8)
	Bachelor’s degree or higher	35 (58)
**Racial identity**		
	American Indian or Alaska Native	1 (1)
	Asian	6 (7)
	Black or African American	39 (44)
	Multiracial	6 (7)
	White	28 (32)
	Other^d^	8 (9)
**Ethnic identity**		
	Hispanic or Latinx	19 (22)

^a^Other sexual orientations as entered by the respondents: pansexual (2/88, 2%), asexual (1/88, 1%), and other (1/88, 1%).

^b^Among deliberants aged >18 years.

^c^GED: General Educational Development.

^d^Other racial identities as entered by the respondents: Hispanic (mixed; 1/88, 1%), Afrolatina (1/88, 1%), Mestizo (1/88, 1%), “none of these races apply to me as I am Hispanic” (1/88, 1%), West Asian (1/88, 1%), and other (1/88, 1%).

#### Facilitators

A total of 18 persons facilitated the deliberations across the 4 sites. The number of facilitators at each site varied according to the size of the deliberant group. Of the 18 facilitators, 8 (44%) were graduate students, 4 (22%) were research staff members, and 6 (33%) were faculty. Their racial and ethnic identities included non-Hispanic White, non-Hispanic Black, Asian, Hispanic, and multiracial; 61% (11/18) were women. The research team and content experts had expertise in HIV prevention, adolescent health, research regulation, bioethics, and public deliberation. The site stakeholders included health care professionals and community advocates. Because the roles that the team members played in the deliberation sessions were fluid (eg, public deliberation experts lead plenary sessions and facilitated breakout group sessions, and scientists from the research team and site stakeholders presented expert information and helped facilitate sessions), for the purpose of this assessment, all team members were considered facilitators.

The facilitators had different training experiences. Of the graduate student facilitators, 75% (6/8) were employed and trained by the deliberation experts, and 25% (2/8) were employed and trained by the research team. Content experts did not receive training in deliberative methods. A booster training session was provided between deliberations at sites 3 and 4; the session focused primarily on methods to support deliberative principles and goals but also included basic group facilitation skills.

### Blueprint for Implementing a Framework for Assessing Facilitator Fidelity

The blueprint report [16] directs researchers to highlight pertinent facilitator remarks on verbatim transcripts of deliberation sessions and rate them as consistent or inconsistent with any of the 6 principles of public deliberations in the framework. This approach is based on the assumption that facilitator remarks consistent with one of the principles encourage deliberant discussions that reflect deliberation ideals, whereas facilitator remarks inconsistent with one of the principles can inhibit such discussions. The principles in the framework are equal participation [23], expression of diverse opinions [24], respect for others [23], adoption of a societal perspective [23], reasoned justification of ideas [23], and compromise or movement toward consensus [24]. We developed a coding rule table that displays the 6 principles, coding rules for each principle, and examples of text coded to each principle. This table can be accessed in the blueprint report [16].

The blueprint report also includes 2 tables to organize codes for analysis: a code display table and a code summary table. In the code display table, transcript line numbers that were coded to the principles are placed in cells defined by the principle, the role of the facilitator who made the remark, and the deliberation session in which the remark was made. In the code summary table, the number of remarks coded as consistent or inconsistent with each principle across all sessions is summarized and displayed. An example of the code display and code summary tables for site 1 (Tampa) can be found in the blueprint report [16].

The blueprint report also provides a step-by-step analytic plan to assess facilitator fidelity at each site [16]. In summary, 2 raters use the coding rule table to independently highlight facilitator remarks on deliberation transcripts that reflect 1 of the 6 principles and indicate whether each remark was consistent or inconsistent with the principle. They compare their coding and resolve discrepancies through discussion and review of transcript data. The transcript line numbers of the agreed-upon codes are placed in the appropriate cell in a code display table. Information in the code display tables for each site is tabulated to then complete a code summary table.

## Results

### Overview

Following the blueprint report [16] guidelines discussed previously, we coded all the transcripts and developed code display and code summary tables for each site. For the project described in this paper, we also developed 2 additional project summary tables to display counts across multiple sites. The first project summary table (Table 2) shows the number of facilitator remarks coded as consistent or inconsistent with the principles by site. The second project summary table (Table 3) shows the number of facilitator remarks coded as consistent or inconsistent with the principles by principle.

Facilitator fidelity to each principle across all sessions and sites can be described based on the project summary tables. For each principle, the total number of remarks coded to that principle, the number of remarks coded as consistent or inconsistent with the principle, and the number of remarks coded to the principle for each site are presented in the following sections. The types of remarks coded to the principles are then discussed, and verbatim examples of facilitator remarks and facilitator and deliberant dialogues are presented. The 3 principles related to facilitation of the discussion (ie, equal participation, expression of diverse opinions, and respect for others) are discussed first followed by the 3 principles related to facilitation of recommendations (ie, adoption of a societal perspective, reasoned justification of ideas, and compromise or movement toward consensus).

**Table 2:** First project summary table—number of facilitator remarks coded as consistent or inconsistent with principles of democratic deliberation by site.

Site and principle			Consistent, n (%)		Inconsistent, n (%)
**Tampa, Florida**					
	Equal participation (n=11)	9 (81.8)		2 (18.1)	
	Expression of diverse opinions (n=41)	36 (87.8)		5 (12.2)	
	Respect for others (n=8)	8 (100)		0 (0)	
	Adoption of a societal perspective (n=6)	4 (66.7)		2 (33.3)	
	Reasoned justification of ideas (n=7)	7 (100)		0 (0)	
	Compromise or movement toward consensus (n=38)	29 (76.3)		9 (23.7)	
	Total (n=111)	93 (83.8)		18 (16.2)	
**Baltimore, Maryland**					
	Equal participation (n=6)	6 (100)		0 (0)	
	Expression of diverse opinions (n=7)	6 (85.7)		1 (14.3)	
	Respect for others (n=2)	2 (100)		0 (0)	
	Adoption of a societal perspective (n=0)	0 (0)		0 (0)	
	Reasoned justification of ideas (n=3)	3 (100)		0 (0)	
	Compromise or movement toward consensus (n=21)	18 (85.7)		3 (14.3)	
	Total (n=39)	35 (89.7)		4 (10.3)	
**Denver, Colorado**					
	Equal participation (n=94)	59 (62.8)		35 (37.2)	
	Expression of diverse opinions (n=41)	24 (58.5)		17 (41.5)	
	Respect for others (n=9)	9 (100)		0 (0)	
	Adoption of a societal perspective (n=14)	6 (42.9)		8 (57.1)	
	Reasoned justification of ideas (n=29)	27 (93.1)		2 (6.9)	
	Compromise or movement toward consensus (n=92)	58 (63)		34 (37)	
	Total (n=279)	183 (65.6)		96 (34.4)	
**Chicago, Illinois**					
	Equal participation (n=74)	64 (86.5)		10 (13.5)	
	Expression of diverse opinions (n=69)	44 (63.8)		25 (36.2)	
	Respect for others (n=8)	8 (100)		0 (0)	
	Adoption of a societal perspective (n=4)	1 (25)		3 (75)	
	Reasoned justification of ideas (n=60)	44 (73.3)		16 (26.7)	
	Compromise or movement toward consensus (n=91)	44 (48.4)		47 (51.6)	
	Total (n=306)	205 (67)		101 (33)	
Total across sites (n=735)			516 (70.2)		219 (29.8)

**Table 3:** Second project summary table—number of facilitator remarks coded as consistent and inconsistent with principles of democratic deliberation by principle.

	Equal participation, n/N (%)			Expression of diverse opinions, n/N (%)			Respect for others, n/N (%)			Adoption of a societal perspective, n/N (%)			Reasoned justification of ideas, n/N (%)			Compromise or movement toward consensus, n/N (%)	
	Consistent	Inconsistent	Consistent		Inconsistent	Consistent		Inconsistent	Consistent		Inconsistent	Consistent		Inconsistent	Consistent		Inconsistent
Tampa, Florida	9/11 (81.8)	2/11 (18.2)	36/41 (87.8)		5/41 (12.2)	8/8 (100)		0/8 (0)	4/6 (66.7)		2/6 (33.3)	7/7 (100)		0/7 (0)	29/38 (76.3)		9/38 (23.7)
Baltimore, Maryland	6/6 (100)	0/6 (0)	6/7 (85.7)		1/7 (14.3)	2/2 (100)		0/2 (0)	0/0 (0)		0/0 (0)	3/3 (100)		0/3 (0)	18/21 (85.7)		3/21 (14.3)
Denver, Colorado	59/94 (62.8)	35/94 (37.2)	24/41 (58.5)		17/41 (41.5)	9/9 (100)		0/9 (0)	6/14 (42.9)		8/14 (57.1)	27/29 (93.1)		2/29 (6.9)	58/92 (63)		34/92 (37)
Chicago, Illinois	64/74 (86.5)	10/74 (13.5)	44/69 (63.8)		25/69 (36.2)	8/8 (100)		0/8 (0)	1/4 (25)		3/4 (75)	44/60 (73.3)		16/60 (26.7)	44/91 (48.4)		47/91 (51.6)
Total	138/185 (74.6)	47/185 (25.4)	110/158 (69.6)		48/158 (30.4)	27/27 (100)		0/27 (0)	11/24 (45.8)		13/24 (54.2)	81/99 (81.8)		18/99 (18.2)	149/242 (61.6)		93/242 (38.4)

### Equal Participation

#### Overview

Across the 4 sites, facilitators made 185 remarks that were coded to the principle of equal participation. A total of 74.6% (138/185) of the remarks were coded as consistent with the principle (encouraged equal participation), and 25.4% (47/185) of the remarks were coded as inconsistent with the principle (discouraged equal participation). The proportions of consistent remarks made at each site were as follows: 6.5% (9/138) in Tampa, 4.3% (6/138) in Baltimore, 42.8% (59/138) in Denver, and 46.4% (64/138) in Chicago. The proportions of inconsistent remarks made at each site were as follows: 4% (2/47) in Tampa, 0% in Baltimore, 74% (35/47) in Denver, and 21% (10/47) in Chicago.

#### Consistent With the Principle

In total, 3 types of remarks were coded as consistent with the principle of equal participation. The remarks (1) conveyed the importance of equal participation, (2) invited individual deliberants or subgroups (eg, youth and parents) to speak, and (3) encouraged more talkative deliberants to allow quieter members time to speak.

Facilitators routinely promoted equal participation by making remarks that emphasized the importance of equal participation. These remarks often occurred when facilitators initially discussed deliberation goals and ground rules. The facilitators emphasized that they valued all perspectives and wished to hear from all deliberants. Facilitators also encouraged equal participation throughout the sessions by frequently asking whether anyone else had something to add. A facilitator in Tampa, for example, said, “Comments from others, anybody?” (session 1).

Facilitators also called on individual deliberants or subgroups of deliberants who had been quiet or who had not contributed to a particular discussion. Facilitators sometimes engaged quiet deliberants by directing questions specifically to them (eg, “[XXXX], what are your thoughts on...?”). Facilitators often encouraged youth deliberants to participate. For example, during a discussion about actions that could be taken to protect youth who self-consent for a study, a facilitator in Chicago, said the following:

Do we have any youth that would like to share an opinion or thoughts here?

Session 4; breakout group 3

In some instances, facilitators encouraged deliberants who had already contributed to a discussion to give quieter members a chance to speak. These requests respected the contributions of more talkative deliberants but provided opportunities for silent or disengaged deliberants to weigh in. A facilitator in Denver, for example, said the following:

So if you’re a person like me who has no problem speaking in front of others, maybe step back once before you make repeat additional comments to others. Whereas if you’re a person who tends to wait a little bit more, if you can, step out of your comfort zone and try to make a comment and just let people know where you’re coming from as early as you feel safe doing so and we would all appreciate that.

Session 1

#### Inconsistent With the Principle

In total, 2 types of remarks were coded as inconsistent with the principle of equal participation. The remarks (1) directed questions toward deliberants who had already spoken on an issue and (2) excused quieter deliberants from speaking.

Some facilitators called on deliberants who had already contributed multiple times instead of on those who had been quiet. A facilitator in Tampa, for example, said the following:

So, before we do that [move to another topic], I thought we’d just give one last comment to the one that—to the person that kicked this part of the discussion off. [XXXX], would you like to say anything else?

Session 4

In some instances, facilitators interacted with a few deliberants who were actively discussing an issue without attempting to draw in other deliberants.

Facilitators sometimes made remarks that served to excuse quieter deliberants from providing comments. In a few instances, facilitators suggested that not everyone needed to contribute to a particular discussion. A facilitator in Denver, for example, said the following:

And this case, it doesn’t have to be everyone that shares...

Session 1; breakout group 2

Facilitators occasionally invited quieter deliberants to share their views but then excused them doing so. A facilitator in Denver, for example, asked deliberants who had not spoken to “move out of their comfort zone” but then said, “We would love it if you can do that verbally but if you can’t, that is okay” (session 4).

### Expression of Diverse Opinions

#### Overview

Across the 4 sites, facilitators made 158 remarks that were coded to the principle of expression of diverse opinions. A total of 69.6% (110/158) of the remarks were coded as consistent with the principle (encouraged expression of diverse opinions), and 30.3% (48/158) of the remarks were coded as inconsistent with the principle (discouraged expression of diverse opinions). The proportions of consistent remarks made at each site were as follows: 32.7% (36/110) in Tampa, 5.5% (6/110) in Baltimore, 21.8% (24/110) in Denver, and 40% (44/110) in Chicago. The proportions of inconsistent remarks made at each site were as follows: 10% (5/48) in Tampa, 2% (1/48) in Baltimore, 35% (17/48) in Denver, and 52% (25/48) in Chicago.

#### Consistent With the Principle

In total, 3 types of remarks were coded as consistent with the principle of expression of diverse opinions. The remarks (1) conveyed the importance of expression of diverse opinions, (2) invited diverse opinions during deliberative discussions, and (3) asked what others who were not represented in the deliberations might think about deliberative topics.

Facilitators routinely encouraged the expression of diverse opinions when initially discussing the purpose and the ground rules of the deliberations. They stressed that conflict and dissent, if done amicably, were important to the deliberative process. Facilitators also emphasized the importance of expressing diverse views throughout the sessions. A facilitator in Chicago, for example, said the following:

And I want to emphasize again that it is really important for us to mention that dissent is absolutely, completely fine here, and disagreement, as long as it’s respectful, is quite helpful. So, please, if you do feel a little differently about any of the actions or any trade-offs than others, please don’t feel like you need to self-censor in any way...

Session 4

Facilitators sometimes invited deliberants to share views that were at odds with views expressed by other deliberants. They often did this by inquiring about “other perspectives” among deliberants. In Baltimore, for example, as the group was reaching a consensus on a recommendation that community partners be involved in the consent process, the facilitator asked the following:

So, I don’t think we want to jump to a show of hands yet, but can I just encourage people to weigh in on the notion of community advocates or community partners, especially if you’ve got concerns about them? If you have concerns about them, it’d be great if you mentioned that and articulated them.

Session 4

Facilitators regularly asked deliberants to surmise how the views of other persons or groups not represented in the deliberation might differ from the deliberants’ views. The facilitators indicated that these questions were aimed at “bringing in as many perspectives as possible.” In Denver, for example, a facilitator said the following:

Do we think that there were any perspectives that might be missing, you know, in our particular group?

Session 2; breakout group 3

The following excerpt from the Tampa deliberation demonstrates how questions about persons not represented in the group can draw out diverse perspectives:

Facilitator: So, I guess in other words, do we think—so I think a good amount of the discussion thus far is some of the group is really coming from similar places. So, for the folks who don’t come from the same angle that members of this group are, are there perspectives out there that are different than what we’re talking about? And what do you think they would say about the conversation we’re having today?

Deliberant: I would say that, of course, there’s perspective that’s very different from this group. I think we're all here for kind of a similar reason. And I think that there are a lot of people out there that do not want their children to have sex education. They do not want their children to have access to birth control. They do not want their children to have access to be able to be in medical research that has to do with HIV or STDs, period. I think there is a large group of parents that would be absolutely freaked out by everything that we've talked about today, you know. So yeah, there's a wide range of comfortableness with this topic.

Session 1; breakout group 1

#### Inconsistent With the Principle

In total, 2 types of remarks were coded as inconsistent with the principle of expression of diverse opinions. The remarks (1) revealed the personal opinions of the facilitators and (2) interrupted the expressions of diverse opinions by deliberants.

We chose to code remarks in which facilitators expressed their own personal opinions as inconsistent with the principle of expression of diverse opinions. Deliberation facilitators are typically dissuaded from expressing their own views on topics germane to the deliberation as this can squelch dissenting or challenging views of deliberants due to power differences between facilitators and deliberants. Although facilitators in our deliberation did not routinely express their own opinions, they did so occasionally. In Tampa, for example, a facilitator said the following:

I don’t know that we’re very respectful of young people. I mean, just to be blunt, like I don’t think we’re respectful of their time, I don’t think we’re respectful of their interests. I think, you know, research—we do cater to adults, and I just don’t think we often were thinking as much about, you know, like you said, interest in accessibility of young people, because we’re—I don’t know, maybe just not cognizant of what those were.

Session 1; breakout group 2

The following excerpt from the Tampa deliberation demonstrates how expression of personal opinions by facilitators can lead deliberants to agree rather than discuss their own views. In this example, a deliberant suggested that school personnel “flag” students who would benefit from or be eligible for a research study. A facilitator comment changed the focus from school personnel “flagging” potentially eligible students to school personnel being well positioned to weigh in on whether a student would be competent to consent:

Deliberant: So, maybe [teachers] just tapping into it in the beginning of the study for identifying people who would be eligible or benefit might be helpful.

Facilitator: Or at least being able to give a comment in terms of their ability to consent and understand.

Deliberant: Yes.

Facilitator: So, I think the teachers really know the kids enough to know whether they will be able to understand the level of nuance that’s required [to consent]. Maybe not with any responsibility related to that, but at least being able to address this question of maturity versus, you know, being able to—

Deliberant: I completely agree. And we, I mean, oftentimes [we] know about the ability for their parents to consent, the ability for their parents—you know, their level of education, how much they understand about the study themselves. I mean, I know my kids pretty well. And I know their families pretty well. I know their living situations. I know a lot about them. So, at least tapping into that perspective, we definitely have a lot to offer in that point of view.

Session 4

In some instances, facilitators inquired about diverse opinions but did not wait for deliberants to comment, and instead, the facilitators answered their own inquiries. In Denver, for example, after a deliberant spoke in favor of community consultation for youth who had no support at home, the facilitator said the following:

What do others think about this? Maybe something perhaps how practical this might be because something like obtaining community input requires a community to show up...So do you think it would realistically work?

Session 4; breakout group 1

### Respect for Others

#### Overview

Across the 4 sites, facilitators made 27 remarks that were coded to the principle of respect for others. All 27 remarks (100%) were coded as consistent with the principle (promoted respect for others). No remarks were coded as inconsistent with the principle (discouraged respect for others). The proportions of consistent remarks made at each site were as follows: 30% (8/27) in Tampa, 7% (2/27) in Baltimore, 33% (9/27) in Denver, and 30% (8/27) in Chicago.

#### Consistent With the Principle

In total, 2 types of remarks were coded as consistent with the principle of respect for others. The remarks (1) conveyed the importance of respect for others and (2) acknowledged instances in which deliberants showed respect for others.

The facilitators routinely stressed the importance of respect for others in their introductory remarks to deliberants. Facilitators emphasized that showing respect for others by actively listening and considering others’ views was a ground rule of the deliberations. A facilitator in Denver, for example, said the following:

We were all experts in our own experience. And when people were speaking about that, the best thing we can do for each other is listen to that and learn from it.

Session 1

Facilitators at times acknowledged when deliberants showed respect for others by pointing out exchanges among deliberants that were particularly respectful. A facilitator in Baltimore, for example, reflected on a previous deliberation:

We have parents, and younger people, and people that have different relationships with their parents, and it was super respectful, and I think kind of collaborative.

Session 3

### Adoption of a Societal Perspective

#### Overview

Across the 4 sites, facilitators made 24 remarks that were coded to the principle of adoption of a societal perspective. A total of 46% (11/24) of the remarks were coded as consistent with the principle (encouraged adoption of a societal perspective), and 54% (13/24) of the remarks were coded as inconsistent with the principle (discouraged adoption of a societal perspective). The proportions of consistent remarks made at each site were as follows: 36% (4/11) in Tampa, 0% in Baltimore, 55% (6/11) in Denver, and 9% (1/11) in Chicago. The proportions of inconsistent remarks made at each site were as follows: 15% (2/13) in Tampa, 0% in Baltimore, 62% (8/13) in Denver, and 23% (3/13) in Chicago.

#### Consistent With the Principle

One type of remark was coded as consistent with the principle of adoption of a societal perspective. This type of remark asked deliberants to consider issues of civic good in their consensus recommendations. Facilitators occasionally introduced the idea that the end goal of research was to benefit society as a whole rather than individual research participants. One facilitator in Chicago, for example, said the following:

Like clinical [practice] really focuses on the individual to improve that individual’s health, whereas clinical research, while there’s often benefit to the individual...the purpose of clinical research is to advance scientific knowledge so we know what’s happening in the population.

Session 1

In a few instances, facilitators asked deliberants to consider what is “good for society” when forming their views. A facilitator in Denver, for example, said the following:

Think about these concerns...from a really broad perspective—that means not just from your specific stakeholder perspective but from a community and researcher perspective.

Session 3; breakout group 5

#### Inconsistent With the Principle

One type of remark was coded as inconsistent with the principle of adoption of a societal perspective. In these instances, facilitators encouraged deliberants to share personal experiences and consider how recommendations would affect them personally without inquiring about the societal perspective. For example, the following excerpt from the Denver deliberation demonstrates a missed opportunity to move a discussion from a deliberant’s personal experience as a parent to a discussion of the greater good:

Deliberant: Me as an adolescent parent, I would be able to trust that if my adolescent made the choice to join the study or like a research that they were doing the right thing. And I think it's also good for them to know like what's going on today, versus them not knowing, like what's going on in the world.

Facilitator: Yeah thanks. And what evidence helped you come to that conclusion? Like obviously that perspective as a parent?

Deliberant: So I work at

local hospital] and we do like birth control and you know, the adolescents are able to consent for themselves without their parents knowing. And I feel like some parents don't talk about like HIV and medications, and what the possibility can be. You know they want to keep their kids hidden from everything and protected from everything. But in our reality if we don't communicate with our adolescents then that's where us as parents get in trouble and our adolescents get in trouble. [Session 3; breakout group 5

### Reasoned Justification of Ideas

#### Overview

Across the 4 sites, facilitators made 99 remarks that were coded to the principle of reasoned justification of ideas. A total of 82% (81/99) of the remarks were coded as consistent with the principle (encouraged reasoned justification of ideas), and 18% (18/99) of the remarks were coded as inconsistent with the principle (discouraged reasoned justification of ideas). The proportions of consistent remarks made at each site were as follows: 9% (7/81) in Tampa, 4% (3/81) in Baltimore, 33% (27/81) in Denver, and 54% (44/81) in Chicago. The proportions of inconsistent remarks made at each site were as follows: 0% in Tampa, 0% in Baltimore, 11% (2/18) in Denver, and 89% (16/18) in Chicago.

#### Consistent With the Principle

In total, 2 types of remarks were coded as consistent with the principle of reasoned justification of ideas. The remarks asked deliberants to (1) provide a reason for their stated views or (2) reveal what information influenced their views.

Some facilitators encouraged deliberants to provide a rationale or explanation for why they took a particular stance on a deliberation topic. In some instances, facilitators discussed the importance of understanding the “why” or the “logic” behind opinions. A facilitator in Tampa, for example, said the following:

We want to give reasons for—in sentences we were writing [consensus recommendations], we want to also say why we think this, not only that, why we think it should be done.

Session 4

Facilitators at times asked deliberants to pinpoint information they had heard in the course of the deliberation or elsewhere that had influenced their opinions. Some facilitators asked deliberants to state what facts supported their views. A facilitator in Baltimore, for example, said the following:

So, I was going to try to nudge the discussion a little bit more towards explanations you’ve heard of current practice or the background reading you did coming in...

Session 4

#### Inconsistent With the Principle

One type of remark was coded as inconsistent with the principle of reasoned justification of ideas. This type of remark encouraged deliberants to justify their opinions but then interrupted them from doing so by supplying a rationale or by excusing the participant from having to explain their reasoning. We did not code remarks in which facilitators assured deliberants that they were free to share their views only if they were comfortable doing so, especially early in the deliberations, but rather remarks in which facilitators invited deliberants to discuss the reasoning for their views but did not give them the opportunity or space to do so.

The following excerpt from the Denver deliberation demonstrates how a facilitator remark can discourage discussion of reasoned justification of ideas. In this example, a deliberant opined that opening a study to cisgender or heterosexual youth would not change their opinion about minor self-consent. The facilitator asked a deliberant to justify their opinion but then excused them from doing so:

Deliberant: And to what [deliberant] said, I think that opening it up to a larger population doesn’t necessarily mean that you’re going to be getting participants from that larger population if they don’t think that it’s an issue that impacts them. Yeah, so I’m not really sure if—how much of an impact it would have for me in terms of like the consent process.

Facilitator: Okay. Thanks. And what would you say helped you come to that conclusion? What would you say is the information that helped you support that idea? And if you don’t want to expand, you don’t have to. So don’t worry. All right everyone. I think this is a great time to move on to the next permutation.

Session 3; breakout group 2

### Compromise or Movement Toward Consensus

#### Overview

Across the 4 sites, facilitators made 242 remarks that were coded to the principle of compromise or movement toward consensus. A total of 61.6% (149/242) of the remarks were coded as consistent with the principle (encouraged compromise and movement toward consensus), and 38.4% (93/242) of the remarks were coded as inconsistent with the principle (discouraged compromise and movement toward consensus). The proportions of consistent remarks made at each site were as follows: 19.5% (29/149) in Tampa, 12.1% (18/149) in Baltimore, 38.9% (58/149) in Denver, and 61.1% (91/149) in Chicago. The proportions of inconsistent remarks made at each site were as follows: 10% (9/93) in Tampa, 3% (3/93) in Baltimore, 37% (34/93) in Denver, and 51% (47/93) in Chicago.

#### Consistent With the Principle

In total, 3 types of remarks were coded as consistent with the principle of compromise or movement toward consensus. The remarks (1) introduced compromise and consensus as a goal of the deliberative process, (2) promoted discussion of common ground and encouraged deliberants to seek consensus, and (3) asked deliberants to summarize the consensus they had reached.

Facilitators discussed the goals of compromise and consensus when making introductory remarks about the deliberation. They informed deliberants that, while they did not need to agree, they were to explore common ground and, if possible, reach consensus on issues that were the focus of the deliberation. In Denver, for example, the facilitator stated the following:

And then in the final session, we’ll review all the actions and tradeoffs that we’ve come up with, see where we have any common ground and collaborate to turn this into an actual list of recommendations or at least guiding principles...

Session 1

Often later in the deliberations, facilitators encouraged participants to consider common ground and move toward consensus. The facilitators asked whether deliberants were “on the same page” about an issue, and if they had not reached a consensus, the facilitators made remarks aimed at encouraging compromise. The facilitators often asked deliberants to consider what “tradeoffs” they would be willing to accept to agree on recommendations. In Baltimore, for example, a facilitator said the following at the start of the last session:

So, now in this phase, in addition to revising or adding or tweaking the things that we were just talking about, the main goal of this next phase is to come up with—there’s different ways to put it, a set of principles or maybe a set of recommendations.

Session 4

While facilitators often summarized their perceptions of deliberant common ground, at times they asked deliberants to discuss the consensus they had reached and recommendations they wished to make. During deliberations in Chicago (the final deliberation site), for example, facilitators asked for volunteers from each breakout group to share a summary of their group’s consensus and recommendations. The volunteers provided comprehensive summaries to the larger group. The following excerpt shows how a deliberant summarized the breakout group’s conclusions, trade-offs, and recommendations related to the use of community town halls:

Facilitator: So, we’re going to try to report out from all our three different groups and kind of keep track of where we had similarities and differences, and then we’ll start to deliberate as a large group as best we can right after that. So, let’s get to it. We’ll start off with group one. I know that the note taker took some notes on the slides, and so—and we have a participant who will be walking us through those.

Deliberant: OK. Yeah. So, as far as like actions and group conclusions, how we feel about a town hall with community before study recruitment begins...as well as like the paid community advisory board that can be engaged at every step along the way. We feel one—it’s useful if the young people do not have support at home. We require honest discussion with the young person as well as like trained professionals to screen. So, not that this wouldn’t be useful for people who have like a more stable and supportive home life but especially important to folks who don’t have that or who don’t have it consistently. A negative is in the community, people [who] aren’t involved with other people that much would not—would like—prefer not to really know about these sorts of things unless like some sort of like major issue popped up. And of course, that varies from like neighborhood to neighborhood but it is kind of like a general thing. Like, at the end of the day, like the majority of the US [are] still like largely conservative, so, yeah. And then c, it could be good but it could be bad like if the broader community were invited. It could attract people who maybe have like more like ill-intent. So, like, you know, the fundamentalist types that come and like, you know, want to disrupt instead of really have a constructive conversation. So, we’d have to just be wary of people who want to show up and just cause dissent and not really like be a part of the conversation. Not—You know, and folks who aren’t really centering the youth at the end of the day, or they may claim to center the youth but it’s really like under the guise of like homophobia, transphobia, hodophobia, all those things. We decided it’s better if we were going to have a community advisory board that it’s a paid group of people who are screened. And we would want to be selective, including people already involved with the youth, such as, you know, leaders and folks working in schools, folks specifically working with like youth HIV prevention, people working within like the queer community who know how to advocate for folks who are—yeah, show up in the range of like queerness and gender and all of these things as well as like near peers. So, people who maybe like not too long ago were going through something similar to this. Yeah. OK...

Session 4

The deliberant continued to discuss a number of other recommendations that the group had agreed on.

#### Inconsistent With the Principle

One type of remark was coded as inconsistent with the principle of compromise or movement toward consensus. In these remarks, facilitators conveyed their “take on” deliberant consensus, often without first asking deliberants what common ground they felt they had reached. Facilitators often provided somewhat lengthy summaries of conclusions reached by breakout groups or by deliberant groups as a whole. These reports eliminated the opportunity for deliberants to consider and articulate points of common ground. The following excerpt from Chicago occurred after deliberant volunteers had reported on the consensus of their breakout groups (as described previously). As the session wound down, the facilitator provided a summary of common ground among the 3 breakout groups based on notes taken by the research team:

Facilitator: Well, in the interest of time, I’m going to do something. I’m going to try to summarize the common ground that we’ve heard...I’m going to try to come up with a summary of what our common ground is that would involve the basic elements of what actions are acceptable under most circumstances and why?...You all see nuance that needs to be added that was in your small groups that we want to make sure we keep in our summarization of your discussion, please keep that in mind and we'll take your revisions right after this. So, I think that we agreed that common ground things that would be necessary or things that would be at least useful in most situations would be community supports such as a town hall and an advisory board. Although there may be some issues with some of these in certain implementation aspects. There was generally support for ongoing support for use throughout this with workshops, counseling during and counseling afterwards. Although people wanted to make sure that it was known while these things were positive, they were not a standalone. So generally, under most circumstances, both those things are good at least options to be part of this. For minor self-consent it sounds like what I was hearing from the group as common ground would be minor self-consent good after the age of 16. And/or in addition to that with teach back as part of it, or on research that does not necessarily have such intense side effects. Or, as one group mentioned, for behavioral, not medical research. General agreement that minor self-consent with adult help is useful, possibly especially for people under 16, but that both this and the trusted adult permission could introduce problems if it becomes in conflict with an adult—with a parent, and potentially for other reasons as well. And then, general agreement that under most situations, an ombudsperson being available as part of the project for all participants—all youth participants, but especially those without—where parental consent isn't feasible was positive. And then, parental permission for folks who were less than 16 in most areas...

Session 4

The facilitator then reported on several other areas of common ground, but the session ended soon after this summary, and there was no opportunity for deliberants to discuss “nuances,” as the facilitator had suggested.

## Discussion

### Principal Findings

The analysis presented in this paper provides an example of how the framework that our team developed to assess facilitator fidelity to principles of democratic deliberation was used in a 4-site public deliberation project focusing on minor self-consent for biomedical HIV prevention research. The assessment was implemented as outlined in our published blueprint report [16]. The examination of facilitator fidelity at all 4 sites allowed us to examine patterns of facilitator remarks across sessions and sites, provided sufficient data to delineate different types of remarks aligned with each principle, and yielded rich verbatim exemplars of facilitator remarks and facilitator and deliberant dialogue reflecting each principle. The counts provide affirmation of facilitator fidelity as most facilitator remarks made across all sessions at all sites were coded as consistent with a principle, and moreover, the number of coded facilitator remarks generally increased over time across the sites.

### Implications

Although the code counts do not serve as definitive quantitative metrics of fidelity as the counts are influenced by variations in the deliberation sites and sessions (eg, number of deliberants attending, structure of sessions, and unique group characteristics), they do provide guideposts for improving deliberation quality. In Table 4, we summarize facilitator fidelity across the 4 sites, provide conclusions about facilitator fidelity across sites, and offer recommendations for future deliberations.

**Table 4:** Summary of facilitator fidelity across sites, conclusions about facilitator fidelity across the 4 sites, and recommendations for future deliberations.

Principle	Summary of facilitator fidelity across the sites	Conclusions about facilitator fidelity across the sites	Recommendations for future deliberations
Equal participation	Few remarks consistent with the principle were made at the first site, and many more were made at the last 2 sites (Tampa: n=9; Denver: n=59; Chicago: n=64). Remarks inconsistent with the principle varied across sites (Tampa: n=2; Denver: n=35; Chicago: n=10).	Remarks consistent with this principle increased after the first 2 deliberations because the research team noted during debriefings that some participants were not well engaged. The team then encouraged facilitators to actively invite quieter members, especially youth, to participate and ask talkative deliberants to pause to give quieter members time to formulate responses. Facilitators also continued on occasion throughout the sessions to encourage persons who had already spoken to comment again or invite quiet deliberants to comment but then immediately assure them that they did not “need” to if they were uncomfortable.	Facilitators should continue to encourage more reticent members to contribute to the discussion and, on occasion, respectfully encourage more talkative members to refrain until others have spoken. Facilitators should be discouraged from inviting deliberants to contribute and then “excuse” them from doing so.
Expression of diverse opinions	Many remarks consistent with the principle were made across the sites (Tampa: n=36; Denver: n=24; Chicago: n=44). Remarks inconsistent with the principle were made with some frequency in later deliberations (Tampa: n=5; Denver: n=17; Chicago: n=25).	Facilitators at all sites reassured participants that differences were valued and dissent was expected or asked them to consider views that might not be represented in their group. However, some facilitators also on occasion continued to give their own opinions rather provide facts or expert information about the issues being discussed.	Deliberants should be encouraged throughout the deliberations to express diverse opinions, and the sharing of personal opinions by facilitators should always be avoided.
Respect for others	Few remarks consistent with the principle were made across the sites (Tampa: n=8; Denver: n=9; Chicago: n=8). No remarks inconsistent with the principle were made across the sites.	There were very few instances in which deliberants did not interact with each other respectfully, and therefore, more facilitator remarks encouraging them to do so were unnecessary.	Once respect for others is established as a ground rule, deliberants may not need ongoing encouragement to act respectfully toward one another, but facilitators should remain alert to and intervene in response to any instances of disrespect.
Adoption of a societal perspective	Few remarks consistent with the principle (Tampa: n=4; Denver: n=6; Chicago: n=1) were made across the sites. Few remarks inconsistent with the principle (Tampa: n=2; Denver: n=8; Chicago: n=3) were made across the sites.	At times, facilitators diverted deliberants’ attention toward their own concerns rather than toward consideration of what would be good for society. The concept of societal benefit, as opposed to self-benefit, might be a difficult concept for some facilitators to understand or promote.	Strategies are needed to help facilitators introduce the concept of societal good and encourage consideration of civic benefit as deliberants finalize their recommendations. While discussion of personal experiences early in deliberations can help members get to know one another, these discussions should not be encouraged later in deliberations as participants move toward consensus.
Reasoned justification of opinions	Remarks consistent with the principle were made often, especially during the last 2 sessions (Tampa: n=7; Denver: n=27; Chicago: n=44). Remarks inconsistent with the principle were made only infrequently (Tampa: n=0; Denver: n=2; Chicago: n=16).	The increase in consistent remarks at the last 2 sites might be attributed to facilitator training as facilitators were encouraged to ask deliberants to expand on how they came to the views they expressed rather than just state their opinions. However, facilitators were less likely to ask deliberants about information that influenced their views. Moreover, infrequently, some facilitators continued to supply reasons for deliberants’ views rather than encouraging them to provide their own rationale.	Facilitators should routinely ask deliberants to discuss the rationale for their views as well as identify what information they considered in coming to these views. Facilitators should never supply their understanding of the reasons or the information underlying deliberant views if the deliberants have not articulated these justifications themselves.
Compromise or movement toward consensus	Many remarks consistent with the principle were made across the sites (Tampa: n=29; Denver: n=58; Chicago: n=44). Remarks inconsistent with the principle were fairly frequent in the last 2 deliberations (Tampa: n=9; Denver: n=34; Chicago: n=47).	Facilitators often encouraged deliberants to consider the views of the group, find common ground, and consider trade-offs but also frequently provided a summary of what the facilitators believed to be a consensus. The increase in consistent remarks in Chicago likely reflected a change in the deliberation plan (run of show) as facilitators were instructed to ask 1 deliberant from each breakout group to assume responsibility for reporting their groups’ points of agreement and recommendations to the larger group. However, the frequency of the inconsistent remarks even late in the deliberations suggests that facilitators continued to discuss what they believed to be the consensus reached by deliberants.	Research teams should develop strategies that allow facilitators to guide deliberants toward compromise and consensus without taking over that responsibility.

### Limitations

While the assessment presented in this paper can inform future deliberation planning and facilitator training, it also raises some issues for further consideration in refining the framework. For example, few facilitator remarks in our deliberation were coded as consistent with the principle of adoption of a societal perspective, and we surmised that this was because some facilitators did not fully understand the concept and, thus, often invited consideration of personal rather than public welfare in the deliberations. This conclusion is challenged by the work of Lehoux and Proulx [25], who suggest that some deliberants can serve as both public and patient representatives. In the former role, they bring a collective perspective, and in the latter role, they bring a personal perspective. In their deliberation on the value of technological health innovations, Lehoux and Proulx [25] found that deliberants alternated between these 2 modes of engagement. When arguing from a collective perspective, they addressed issues such as social costs, environmental protection, and individual freedoms, whereas when they argued from a personal perspective, they addressed issues such as the functionality of the technology, its dehumanizing effects on users, and its potential to increase individuals’ autonomy. The researchers suggest that these 2 engagement modes are complementary and each is needed to advance deliberative discussions. They recommend that facilitators can first elicit one standpoint and then elicit counterpoints from the other standpoint. This work suggests that the facilitator fidelity framework may need to provide a way for researchers to evaluate the extent to which facilitators are able to elicit discussion of both perspectives and illuminate the tension between them rather than rate questions about personal experiences and needs as inconsistent with the principle of adoption of a societal perspective.

Another issue not sufficiently addressed in the framework is plurality. The framework includes the principles of expression of diverse opinions and compromise and movement toward consensus but does not call for an assessment of how facilitators unearth and account for multiple perspectives—despite the fact that deliberants are chosen for their diverse views. In our assessment, remarks coded as inconsistent with the principle of expression of diverse opinions were primarily personal opinions expressed by the facilitators, and remarks coded as inconsistent with the principle of compromise and movement toward consensus were primarily remarks that revealed the facilitators’ “takes” on deliberant points of agreement. While these were important findings and led to recommendations for facilitator training, the nuances of effectively managing plurality are not captured completely in the framework. Baker et al [24] addressed the question of “what to do when people disagree” in deliberations and discussed how different perspectives should be elicited, deliberated, and integrated into recommendations. They developed a multilevel framework that allows for transparency of plurality and considers the integration of perspectives through counting (ie, strength of preferences), coherence (ie, the logical consistency of the argument), and consensus (ie, recommendations agreed upon through deliberation). They propose that agreements and disagreements occur at 3 levels: principles (ie, high-level normative statements), policies (ie, midlevel operational rules), and patients (ie, case-level judgments). Plurality can be disentangled by examining counting, consensus, and coherence at each level. For example, deliberants may reach consensus on general principles, but these principles may not be reflected in their policy recommendations, and thus, their decision-making lacks coherence. The authors suggest that facilitation methods can be developed to elicit values at all levels and uncover instances in which principles, policies, and patients are in tension, thereby promoting discussions of inconsistencies, identification of values that have the greatest force, and increased justification of collective outputs. This work could inform the refinement of our framework by introducing important nuances in our evaluation of how facilitators elicit diverse opinions and guide deliberants to integrate multiple perspectives into their final recommendations.

The issue of facilitator influence raised in our assessment is considered in a discussion by Kuhar et al [26]. We identified a few exchanges in which facilitators expressed their opinions, which in turn influenced deliberant responses. Kuhar et al [26] point out that neutrality of facilitators is a deliberation ideal but facilitator influence on outcomes occurs regularly in deliberative discussions. We recognize that, while our framework classified a few overt instances of facilitator influence, it is possible that facilitators influenced discussions in more nuanced ways not addressed in the framework (eg, information presented in expert testimonies), suggesting that facilitator neutrality is an issue that warrants further development. For example, assessment of facilitators’ performance might include the extent to which they have reflected on their own views and developed an awareness of how their views can influence deliberation outcomes, even if subtly.

In addition to the limitations described previously, a few other concerns need to be addressed in the refinement of the framework. Kuhar et al [26] pose that the privileging of logical argumentation, which is central to most deliberation theories but not valued by all cultures, can marginalize underrepresented groups. In future iterations of our framework, we intend to add a principle that extends beyond expression of diverse perspectives and addresses mitigating social inequalities more explicitly. In addition, for the assessment reported in this paper, all team members (eg, deliberation experts, research team members, and site stakeholders) were treated as one group of facilitators as there was fluidity in how they contributed to the sessions. However, in future assessments, we will examine role differences more closely when determining how facilitation processes overall affect deliberation quality.

### Conclusions

Our assessment of facilitator fidelity to the principles of democratic deliberation in a 4-site deliberation project on the acceptability of youth self-consent for biomedical HIV prevention research affirmed that most facilitator remarks aligned with deliberative principles but also revealed areas in which facilitator performance could be strengthened. Facilitators play an important role in ensuring rich deliberative discussions, and a comprehensive assessment of deliberation quality should include an assessment of facilitator performance. The facilitator fidelity framework and blueprint report developed by our team offer a guide to conduct such an assessment. Systematically assessing facilitator fidelity can not only provide an indication of deliberation quality but can also inform future deliberation planning and facilitator training.

## Abbreviations

IRB: institutional review board

## Data Availability

The datasets generated and analyzed during this study are not publicly available, nor are they available from the corresponding author on reasonable request. The Patient-Centered Outcomes Research Institute determines which of its awards meet the criteria for inclusion in its data repository. The terms of the award that supported this study *do not* require participation in the data repository. Much of the information discussed in the deliberations was very sensitive in nature, and the small number of research participants would make it difficult to protect the privacy of their sensitive disclosures in the sessions. For these reasons, the full transcripts will not be made available.

## References

[1] Blacksher E, Hiratsuka VY, Blanchard JW, Lund JR, Reedy J, Beans JA, Saunkeah B, Peercy M, Byars C, Yracheta J, Tsosie KS, O'Leary M, Ducheneaux G, Spicer PG (2021). Deliberations with American Indian and Alaska native people about the ethics of genomics: an adapted model of deliberation used with three tribal communities in the United States. AJOB Empir Bioeth.

[2] Scherer M, Kamler A, Weiss L, Blacksher E, Jeavons J, Gold MR (2022). Using public deliberation to set priorities: the case of COVID-19 vaccine access in New York City. J Public Health Manag Pract.

[3] Slomp C, Edwards L, Burgess M, Sapir-Pichhadze R, Keown P, Bryan S (2023). Public values and guiding principles for implementing epitope compatibility in kidney transplantation allocation criteria: results from a Canadian online public deliberation. BMC Public Health.

[4] Hiratsuka VY, Beans JA, Blanchard JW, Reedy J, Blacksher E, Lund JR, Spicer PG (2020). An Alaska Native community's views on genetic research, testing, and return of results: results from a public deliberation. PLoS One.

[5] Richmond J, Mangrum R, Wang G, Maurer M, Sofaer S, Yang M, Carman KL (2019). An informed public's views on reducing antibiotic overuse. Health Serv Res.

[6] Jagsi R, Griffith KA, Jones RD, Krenz C, Gornick M, Spence R, De Vries R, Hawley ST, Zon R, Bolte S, Sadeghi N, Schilsky RL, Bradbury AR (2019). Effect of public deliberation on patient attitudes regarding consent and data use in a learning health care system for oncology. J Clin Oncol.

[7] Waljee AK, Ryan KA, Krenz CD, Ioannou GN, Beste LA, Tincopa MA, Saini SD, Su GL, Arasim ME, Roman PT, Nallamothu BK, De Vries R (2020). Eliciting patient views on the allocation of limited healthcare resources: a deliberation on hepatitis C treatment in the Veterans Health Administration. BMC Health Serv Res.

[8] Schwartz PH, O'Doherty KC, Bentley C, Schmidt KK, Burgess MM (2021). Layperson views about the design and evaluation of decision aids: a public deliberation. Med Decis Making.

[9] Raj M, Ryan K, Nong P, Calhoun K, Trinidad MG, De Vries R, Creary M, Spector-Bagdady K, Kardia SL, Platt J (2022). Public deliberation process on patient perspectives on health information sharing: evaluative descriptive study. JMIR Cancer.

[10] Trinidad SB, Blacksher E, Woodbury RB, Hopkins SE, Burke W, Woodahl EL, Boyer BB, Hiratsuka VY (2022). Precision medicine research with American Indian and Alaska Native communities: results of a deliberative engagement with tribal leaders. Genet Med.

[11] Scott AM, Sims R, Degeling C, Carter S, Thomas R (2019). Developing and applying a deductive coding framework to assess the goals of citizen/community jury deliberations. Health Expect.

[12] Goold SD, Biddle AK, Klipp G, Hall CN, Danis M (2005). Choosing Healthplans All Together: a deliberative exercise for allocating limited health care resources. J Health Polit Policy Law.

[13] Rubinelli S, von Groote PM (2017). Stakeholder dialogue as deliberation for decision making in health policy and systems: the approach from argumentation theory. Am J Phys Med Rehabil.

[14] Dillard KN (2013). Envisioning the role of facilitation in public deliberation. J Appl Commun Res.

[15] De Vries R, Stanczyk AE, Ryan KA, Kim SY (2011). A framework for assessing the quality of democratic deliberation: enhancing deliberation as a tool for bioethics. J Empir Res Hum Res Ethics.

[16] Draucker C, Carrión A, Ott MA, Knopf A (2023). Assessing facilitator fidelity to principles of public deliberation: tutorial. JMIR Form Res.

[17] Community solutions to adolescent research consent - minor consent for biomedical HIV research (C-START). National Institutes of Health National Library of Medicine.

[18] Do public deliberation methods help improve the informed consent process for research on sensitive topics?. Patient-Centered Outcomes Research Institute.

[19] (1977). Research involving children: report and recommendations. U.S. Department of Health, Education and Dhew Publication.

[20] CFR - Code of federal regulations title 21. U.S. Food & Drug Administration.

[21] Arrington-Sanders R, Wilson CM, Perumean-Chaney SE, Patki A, Hosek S (2019). Brief report: role of sociobehavioral factors in subprotective TFV-DP levels among YMSM enrolled in 2 PrEP trials. J Acquir Immune Defic Syndr.

[22] Ott MA, Knopf AS (2019). Avoiding a tyranny of the majority: public deliberation as citizen science, sensitive issues, and vulnerable populations. Am J Bioeth.

[23] De Vries R, Stanczyk A, Wall IF, Uhlmann R, Damschroder LJ, Kim SY (2010). Assessing the quality of democratic deliberation: a case study of public deliberation on the ethics of surrogate consent for research. Soc Sci Med.

[24] Baker R, Mason H, McHugh N, Donaldson C (2021). Public values and plurality in health priority setting: what to do when people disagree and why we should care about reasons as well as choices. Soc Sci Med.

[25] Lehoux P, Proulx S (2019). Deliberating as a public representative or as a potential user? Two complementary perspectives that should inform health innovation policy. Healthc Policy.

[26] Kuhar M, Krmelj M, Petrič G (2019). The impact of facilitation on the quality of deliberation and attitude change. Small Group Res.

